# Author Correction: Ultra-high-granularity detector simulation with intra-event aware generative adversarial network and self-supervised relational reasoning

**DOI:** 10.1038/s41467-024-49971-x

**Published:** 2024-07-11

**Authors:** Baran Hashemi, Nikolai Hartmann, Sahand Sharifzadeh, James Kahn, Thomas Kuhr

**Affiliations:** 1 ORIGINS Data Science Lab, Technical University Munich, Munich, Germany; 2 Faculty of Physics, Ludwig Maximilians University in Munich, Munich, Germany; 3 Faculty of Computer Science, Ludwig Maximilians University in Munich, Munich, Germany; 4 Helmholtz AI, Karlsruhe, Germany; 5 Steinbuch Centre for Computing (SCC), Karlsruhe Institute of Technology (KIT), Karlsruhe, Germany

**Keywords:** Experimental particle physics, Computer science

Correction to: *Nature Communications* 10.1038/s41467-024-49104-4, published online 08 June 2024

In this article the affiliation details for Thomas Kuhr and Nikolai Hartmann were incorrectly given as ‘ORIGINS Data Science Lab, Technical University Munich, Munich, Germany’ but should have been ‘Faculty of Physics, Ludwig Maximilians University in Munich, Munich, Germany’. The original article has been corrected.

