# Supplementary material for: Ultra-high-granularity detector simulation with intra-event aware generative adversarial network and self-supervised relational reasoning
Source: Nat Commun. 2024 Jun 8;15:4916. doi: 10.1038/s41467-024-49104-4 (PMC11162486; doi:10.1038/s41467-024-49104-4)
Supplement: Supplementary file 1 — Supplementary Information [file 41467_2024_49104_MOESM1_ESM.pdf]

Supplementary Note for
Ultra-High-Resolution Detector Simulation with Intra-Event Aware Generative Adversarial Network and Self-Supervised Relational Reasoning

Baran Hashemi[*1], Nikolai Hartmann[†1], Sahand Sharifzadeh[‡2], James Kahn[§3,4], and Thomas Kuhr[¶1]

[1]Faculty of Physics, Ludwig Maximilians University in Munich, Germany
[2]Faculty of Computer Science, Ludwig Maximilians University of Munich, Germany
[3]Helmholtz AI, Germany
[4]Steinbuch Centre for Computing (SCC), Karlsruhe Institute of Technology (KIT), Germany

1 Ablation Studies and Things We Tried But Did Not Work

For the IEA-loss we tested several losses in order to achieve the best stability, shown in Figure 1. Some of them have their own merits and downsides. We explored the Kullback-Leibler (KL) divergence [1], the L1 loss, the Huber loss [2], and the L2 loss. KL divergence was more stable in capturing differences between the real and fake self-similarities and more robust in outliers.

We probed a range of coefficients for the IEA-loss and the Uniformity loss. For the KL divergence as the IEA-loss, we tried the values $\{0.1, 1, 5, 10\}$ and selected 1. For the L1 loss, as well as the IEA-loss, the best λ_{IEA} value is 10. For the Uniformity loss, we probed the values $\{0.01, 0.1, 0.5, 0.75, 1, 5, 10\}$ and selected 0.1. Moreover, IEA-GAN, without the IEA-loss and Uniformity loss, suffers from the lack of agreement maximization penalty for the generator and information maximization for the discriminator. Our study shows that having either of these losses without the other causes training instability, divergence, and lower fidelity, as shown in Table 1.

*baran.hashemi@origins-cluster.de
†nikolai.hartmann@physik.uni-muenchen.de
‡sharifzadeh@google.com
§james.kahn@kit.edu
¶thomas.Kuhr@lmu.de

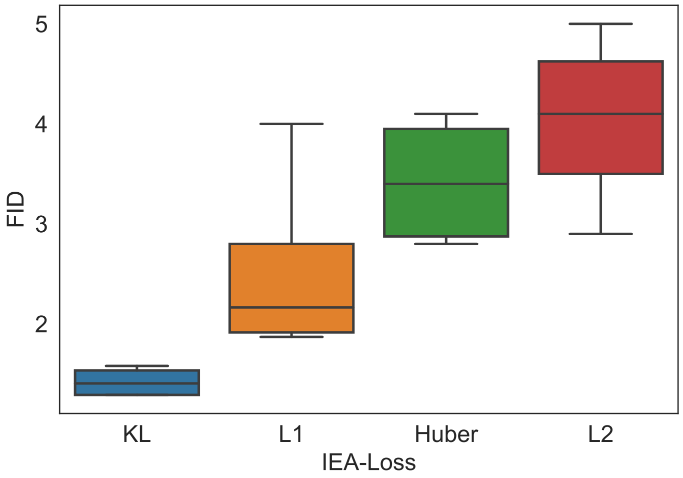

Supplementary Figure 1: Comparison of the FID between different IEA-losses

Supplementary Table 1: FID comparison between IEA-GAN, IEA-GAN with RRM only, IEA-GAN with Uniformity loss only, and IEA-GAN with both IEA-loss, averaged across six random seeds.

	IEA-GAN	Only RRM	RRM with Uniformity	RRM with IEA-loss
**FID**	$\mathbf{1.50 \pm 0.16}$	2.74 ± 0.62	2.29 ± 0.14	3.42 ± 0.52

For the hypersphere dimension, we probed the values {512, 768, 1024, 2048} and selected 1024. For dimensions smaller than 512, the discriminator fails to converge. We also changed the position of the hypersphere projection layer and put it before and after the Multi-head attention [3]. The best position for the hypersphere projection is after the Multi-head attention and two layers of MLP. Moreover, for the hypersphere projection, we also tried an inverse Stereographic Projection $h : \mathbb{R}^N \rightarrow \mathbb{S}^N/\{p\}$ with p as a north pole on the n-sphere [4] instead of L2 compactification. This map is conformal; thus, it locally preserves angles between the data points. The results were more stable, but the average FID was better with L2 compactification, as shown in Table 2.

Supplementary Table 2: FID comparison between two different Hypersphere projections for IEA-GAN's discriminator, averaged across six random seeds.

	L2 compactification	Inverse Stereographic projection
**FID**	$\mathbf{1.50 \pm 0.16}$	2.01 ± 0.07

Inside the RRM, we tried a GeLU [5] non-linearity instead of ReLU, and the result was in favor of the latter. We also put the layer normalization before and after the Multi-head Attention. The pre-norm version seems to be much more stable and adaptable to GAN [6] training intricacies. Another observation related to RRM is the weight normalization of the linear layers. We observed that for the discriminator, spectrally normalized MLPs show the best results. For the Generator, applying Spectral Normalization [7] to the linear layers destabilizes the training. Our observation regarding the effect of RRM over the generator's label embedding shows that without it, the RRM in the discriminator

also becomes unstable, and the training diverges very early.

For the random degrees of freedom (Rdof), first, we utilized the random vectors that are fed to the generator and applied the RRM on top of it. However, the FID [8] did not reach values below 20, and there was no correlation. Hence, we introduced separate random sampling for event generation for which we probed dimensions {2, 4, 8, 16, 32}. 4 degrees of freedom was the most optimal choice. We observed that as the dimension of Rdof increases, the intra-event correlation fades away between the generated images. We also checked the Uniform distribution for event Rdof sampling, which did not lead to any stable result. Several ways to fuse the Rdof to the class embeddings were tested such as learnable neural network layer (matrix factorization), concatenating, summing, and having an MLP with non-linear part, but eventually chose a learnable neural network layer (matrix factorization) for the feature mixing layer.

We also looked at different combinations of learning rates for G and D. Using the TTUR regime results in a severe mode collapse. Thus, we used the same learning rates for both G and D. We swept through $\{1 \times 10^{-5}, 2.5 \times 10^{-5}, 5 \times 10^{-5}, 7.5 \times 10^{-5}, 1 \times 10^{-4}\}$ and selected 5×10^{-5}. For the backbone model, the shallow version of BigGAN-deep [9], BigGAN [9], led to mode-collapse; therefore, we chose BigGAN-deep.

2 Supplementary Figures and Supplementary Tables

The following fig. 2 is referenced in the Introduction, table 3, is mentioned in the Results, and the Algorithm algorithm 1 is highlighted in Methods section of the main manuscript.

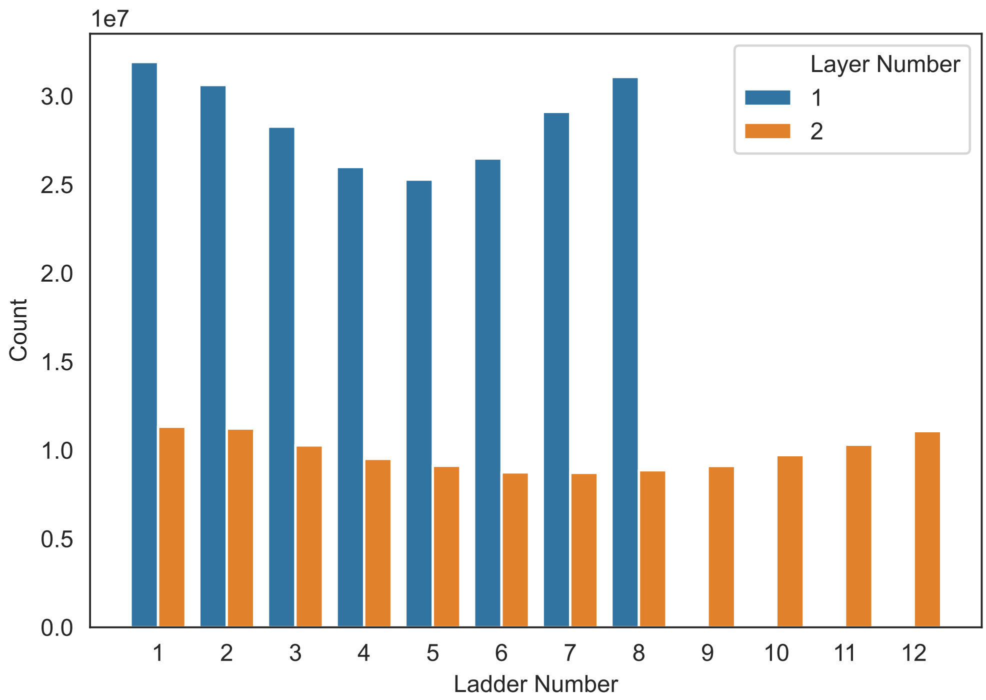

Supplementary Figure 2: The number of PXD hits per layer and sensor (ladder). Each sensor has different occupancies for sensors in the inner and outer layers. The global asymmetry between them stems from the ϕ dependency of PXD images.

Supplementary Table 3: FID Score on PXD images after different jittering methods applied to them, providing interoperability to it's value.

**Image Jitterings**	**FID**
None	2.4×10^{-5}
Random Masking (dead zones)	14.58
Random Noise	87.23
Random Rotation (30 degrees)	23.69
Random Rotation (10 degrees)	2.81
Random Translation (0.1, 0.1)	1.99
Random Shear (10, 10)	23.53
Random Zoom	9.06
High-Intensity Charge Smearing	3.16
Low-Intensity Charge Smearing	47.24

Algorithm 1 Intra-Event Aware GAN

Require: generator and discriminator parameters θ_G, θ_D, Intra-Event-aware coefficient λ_{IEA}, Uniformity coefficient λ_{uniform} and hyperparameter s, Adam hyperparameters α, β_1, β_2, event size M, number of discriminator iteration steps per generator iteration N_D

for number of training iterations **do**
    for $t = 1, ..., N_D$ **do**
        sample $\{\mathbf{z}^i\}_{i=1}^{M} \sim \mathrm{p}(\mathbf{z})$,
        $\{\mathbf{x}^i, \mathbf{y}^i\}_{i=1}^{M} \sim \mathrm{p}_{\text{event}}(\mathbf{x}, \mathbf{y})$, $\{\mathbf{r}^i\}_{i=1}^{M} \sim \mathrm{p}_{\text{Rdof}}(\mathbf{z})$ ▷ Event Sampling.
        for $i = 1, ..., M$ **do**
            $\ell^{(i)}_{\text{D}_{\text{hinge}}} \leftarrow \ell_{\text{D}_{\text{hinge}}}(\mathbf{x}^{(i)}; \mathrm{G}(\mathbf{z}^i, \mathbf{y}^i, \mathbf{r}^i))$
        end for
        $\mathcal{L}_{\text{D}_{\text{hinge}}} \leftarrow \frac{1}{M}\sum_{i=1}^{M} \ell^{(i)}_{\text{D}_{\text{hinge}}}$
        $\mathcal{L}_{\text{uniform}} \leftarrow \mathcal{L}_{\text{uniform}}(\mathbf{x}; s)$ ▷ The Uniformity Loss.
        $\mathcal{L}^{\text{real}}_{2C} \leftarrow \frac{1}{M}\sum_{i=1}^{M} \ell_{2C}(\mathbf{x}^i, \mathbf{y}^i)$
        $\theta_D \leftarrow Adam(\mathcal{L}_{\text{D}_{\text{hinge}}} + \lambda_{2C}\mathcal{L}^{\text{real}}_{2C} + \lambda_{\text{uniform}}\mathcal{L}_{\text{uniform}}, \alpha, \beta_1, \beta_2)$
    end for
    sample $\{\mathbf{z}^i\}_{i=1}^{M} \sim \mathrm{p}(\mathbf{z})$,
    sample $\{\mathbf{r}^i\}_{i=1}^{M} \sim \mathrm{p}_{\text{Rdof}}(\mathbf{z})$ ▷ Event Sampling.
    for $i = 1, ..., M$ **do**
        $\ell^{(i)}_{\text{G}_{\text{hinge}}} \leftarrow \ell_{G_{\text{hinge}}}(\mathrm{G}(\mathbf{z}^i, \mathbf{y}^i, \mathbf{r}^i))$
    end for
    $\mathcal{L}_{\text{G}_{\text{hinge}}} \leftarrow \frac{1}{M}\sum_{i=1}^{M} \ell^{(i)}_{\text{G}_{\text{hinge}}}$
    $\mathcal{L}_{\text{IEA}} \leftarrow \frac{1}{M}\sum_{i=1}^{M} \ell_{\text{IEA}}(\mathrm{G}(\mathbf{z}^i, \mathbf{y}^i, \mathbf{r}^i), \mathbf{x}^i)$ ▷ The Intra-Event Aware Loss.
    $\mathcal{L}^{\text{fake}}_{2C} \leftarrow \frac{1}{M}\sum_{i=1}^{M} \ell_{2C}(\mathrm{G}(\mathbf{z}^i, \mathbf{y}^i, \mathbf{r}^i), \mathbf{y}^i)$
    $\theta_G \leftarrow \mathrm{Adam}(\mathcal{L}_{\text{G}_{\text{hinge}}} + \lambda_{2C}\mathcal{L}^{\text{fake}}_{2C} + \lambda_{\text{IEA}}\mathcal{L}_{\text{IEA}}, \alpha, \beta_1, \beta_2)$
end for

References

[1] Kullback, S., Leibler, R.A.: On Information and Sufficiency. The Annals of Mathematical Statistics **22**(1), 79–86 (1951). https://doi.org/10.1214/aoms/1177729694

[2] Huber, P.J.: Robust Estimation of a Location Parameter. The Annals of Mathematical Statistics **35**(1), 73–101 (1964). https://doi.org/10.1214/aoms/1177703732

[3] Vaswani, A., Shazeer, N., Parmar, N., Uszkoreit, J., Jones, L., Gomez, A.N., Kaiser, Ł., Polosukhin, I.: Attention is all you need. In: Proceedings of the 31st International Conference on Neural Information Processing Systems. NIPS'17, pp. 6000–6010. Curran Associates Inc., Red Hook, NY, USA (2017)

[4] Eybpoosh, K., Rezghi, M., Heydari, A.: Applying inverse stereographic projection to manifold learning and clustering. Applied Intelligence **52**(4), 4443–4457 (2022). https://doi.org/10.1007/s10489-021-02513-0

[5] Hendrycks, D., Gimpel, K.: Gaussian Error Linear Units (GELUs). arXiv. Comment: Trimmed version of 2016 draft (2023). https://doi.org/10.48550/arXiv.1606.08415

[6] Mirza, M., Osindero, S.: Conditional Generative Adversarial Nets. arXiv (2014). https://doi.org/10.48550/arXiv.1411.1784

[7] Miyato, T., Kataoka, T., Koyama, M., Yoshida, Y.: Spectral Normalization for Generative Adversarial Networks. arXiv. Comment: Published as a conference paper at ICLR 2018 (2018). https://doi.org/10.48550/arXiv.1802.05957

[8] Heusel, M., Ramsauer, H., Unterthiner, T., Nessler, B., Hochreiter, S.: GANs trained by a two time-scale update rule converge to a local nash equilibrium. In: Proceedings of the 31st International Conference on Neural Information Processing Systems. NIPS'17, pp. 6629–6640. Curran Associates Inc., Red Hook, NY, USA (2017)

[9] Brock, A., Donahue, J., Simonyan, K.: Large Scale GAN Training for High Fidelity Natural Image Synthesis. arXiv (2019). https://doi.org/10.48550/arXiv.1809.11096
